# Transfemoral Transcatheter Tricuspid Valve Replacement Using the EVOQUE System in an Octogenarian for Severe Tricuspid Valve Regurgitation

**DOI:** 10.7759/cureus.45848

**Published:** 2023-09-24

**Authors:** Besart Cuko, Massimo Baudo, Julien Ternacle, Lionel Leroux, Thomas Modine

**Affiliations:** 1 Department of Cardiology and Cardio-Vascular Surgery, Hopital Cardiologique de Haut-Leveque, Pessac, FRA; 2 Department of Cardiac Surgery, Azienda Socio Sanitaria Territoriale (ASST) degli Spedali Civili di Brescia, University of Brescia, Brescia, ITA

**Keywords:** transcatheter tricuspid valve, transcatheter approach, transcatheter valve replacement, transcatheter intervention, evoque, tricuspid prosthesis, tricuspid valve insufficiency, tricuspid valve regurgitation

## Abstract

Tricuspid regurgitation is a frequent condition that is linked to an elevated risk of cardiovascular events and significant mortality but is often overshadowed by left-sided valve diseases. Isolated surgical tricuspid valve surgery is still considered a high-risk surgery, and over recent years, various transcatheter procedures for tricuspid treatment have emerged as an alternative solution. Among the available transcatheter procedures, the EVOQUE system's transcatheter tricuspid valve replacement could potentially offer a solution, especially in patients considered non-eligible for transcatheter edge-to-edge tricuspid valve repair. We present a case report of an octogenarian patient considered at prohibitive risk for conventional surgery and not eligible for transcatheter edge-to-edge repair who was eventually treated with a transfemoral transcatheter tricuspid 52-mm EVOQUE valve implantation. Postprocedural recovery and follow-up at 18 months were uneventful, with a well-functioning tricuspid valve bioprosthesis.

## Introduction

Tricuspid regurgitation (TR) is a frequent condition, as described by several epidemiological studies [1,2]. Despite being often overshadowed by left-sided valve diseases, TR is linked to an elevated risk of cardiovascular events and significant mortality [3-5]. Even with constant improvement in surgical techniques and post-operative management, isolated surgical tricuspid valve surgery is still considered a high-risk surgery [6]. Over recent years, various transcatheter procedures for tricuspid treatment have emerged, with transcatheter edge-to-edge repair (TEER) being the most prevalent technique, demonstrating both safety and reasonable effectiveness in reducing TR and improving clinical function [7-9]. However, TEER has strict criteria and is not always feasible, leaving a group of patients untreated or with significant residual TR. The EVOQUE system's transcatheter tricuspid valve replacement (TTVR) via transfemoral approach could potentially offer a solution for this patient category. We present a case report of an 82-year-old woman considered at prohibitive risk for conventional surgery and not eligible for TEER who was eventually treated with a transfemoral transcatheter tricuspid 52-mm EVOQUE valve implantation.

## Case presentation

**Figure 1:**
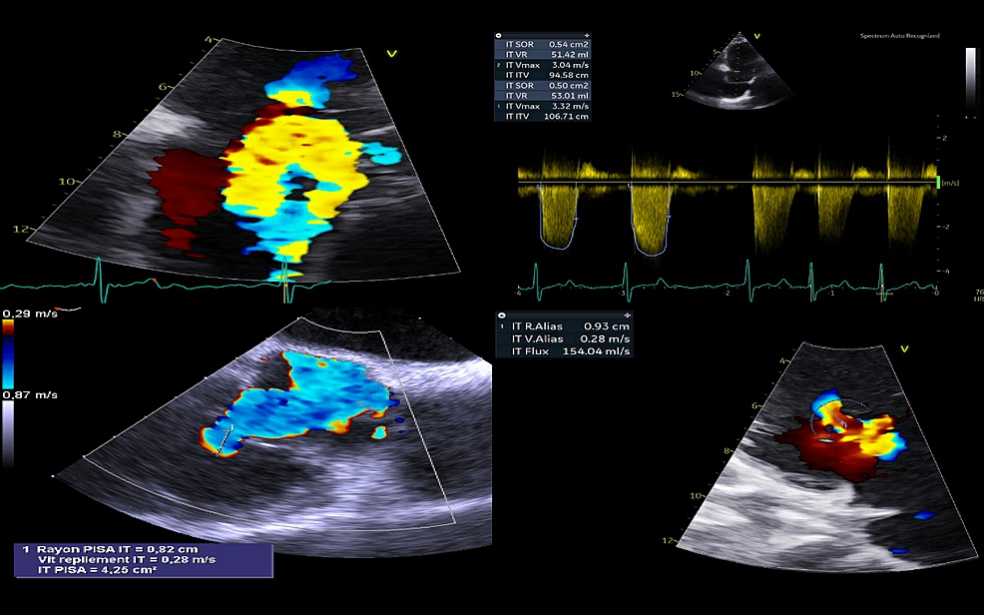
Pre-operative echocardiogram images showing severe tricuspid valve regurgitation

An 82-year-old female was admitted to our institution for signs of right heart failure associated with hepatomegaly and ascites. Her history included permanent atrial fibrillation, breast cancer, and hypothyroidism. Transthoracic and transesophageal echocardiograms showed severe functional TR with tricuspid annular dilatation, right and left atrial dilatation, right ventricular dilatation, and mild dysfunction, while left ventricular geometry and function were preserved (Figure 1). 

After the heart team assessment, the patient underwent transfemoral TTVR. The patient's informed consent for the procedure and data collection for research purposes was obtained. 

**Figure 2:**
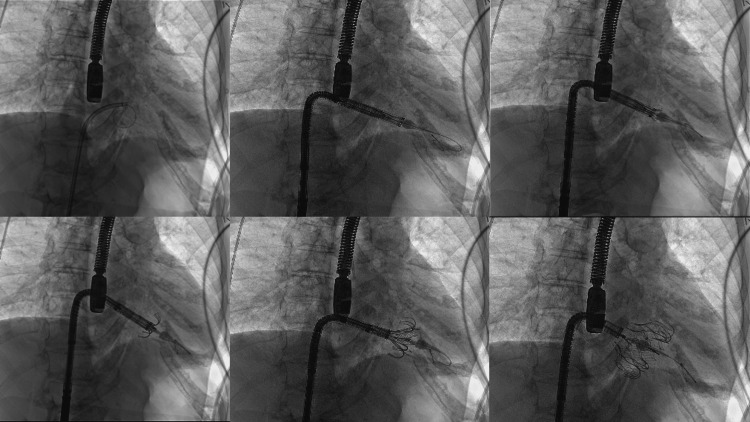
Procedural sequence of transcatheter tricuspid EVOQUE valve implantation

The procedure was performed in a hybrid operating room under general anesthesia. Intravenous unfractionated heparin was given intra-operatively to achieve an activated clotting time (ACT) longer than 250 seconds. Through the right femoral vein, the 28-F EVOQUE system (tricuspid EVOQUE transcatheter heart valve) was advanced across the tricuspid valve. Under strict transesophageal and fluoroscopic guidance, a 52-mm EVOQUE valve (Edwards Lifesciences) was implanted with the immediate elimination of severe TR and a mean gradient of 2 mmHg at the end of the procedure. Then, percutaneous access hemostasis was achieved by using a pre-closure technique with the suture-mediated Proglide device. Total procedure time was 80 min (Figure 2). 

**Figure 3:**
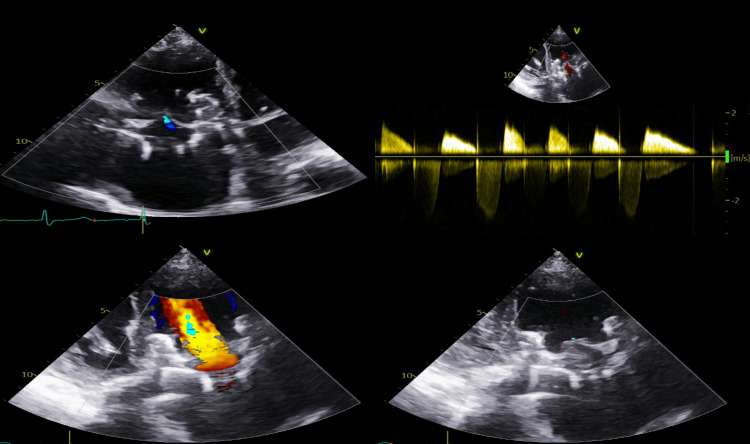
Echocardiogram images at follow-up showing a well-functioning transcatheter tricuspid valve bioprosthesis

Postprocedural recovery was uneventful with a good hemodynamic response. After starting therapeutic anticoagulation, the patient was discharged home three days later. The 18-month follow-up showed a total recovery of New York Heart Association (NYHA) functional class and a well-functioning transcatheter tricuspid valve bioprosthesis with no residual TR or paraprosthetic leak at transthoracic echocardiogram (Figure 3). 

## Discussion

Overall, moderate or severe tricuspid regurgitation prevalence is estimated to range from 0.55% up to 3% after 75 years of age [2]. In spite of its high occurrence, TR frequently goes untreated, even if it's associated with unfavorable results [10,11]. Medical therapy remains the first-line treatment of choice, as most TRs are generally well tolerated. Severe TR refractory to medical management rarely occurs, but when disabling TR appears, surgery is needed. However, handling isolated severe TR is conflicting, often resulting in an initial lack of referral for interventions, thus letting the patient's condition advance. As reported in literature, in-hospital mortality of isolated tricuspid valve surgery is near 10% [6,12]. The significant in-hospital mortality seen in isolated tricuspid valve surgery has paved the way for emerging lower-risk transcatheter solutions for patients with TR and right-sided heart failure [6]. The most commonly used technique is TEER, considered safe and effective with sustained clinical improvement [8,9,13]. Yet, in certain forms of TR, this approach is impractical, or the chances of procedural unsuccessful outcomes are elevated. This is attributed to various factors that predict procedural failure, including baseline massive or torrential TR, notable leaflet tethering, and a coaptation gap >7 mm [14]. Anyway, these indicators of potential failure resemble the ones taken into account in traditional surgical repairs, and those exhibiting these underlying predictors end up undergoing surgical tricuspid valve replacement [15].

TTVR using the EVOQUE valve implantation has emerged as a new treatment option in patients with severe or greater TR. The initial application of the EVOQUE valve replacement system was in mitral valve replacement, and subsequently, it was successfully employed in TTVR [16]. The system consists of a self-expanding nitinol frame, bovine pericardial leaflets, and a fabric skirt to minimize paravalvular leaks. The valve has a unique anchoring mechanism that uses the annulus, leaflets, and chords for stable implantation with nine ventricular anchors [14]. Different studies have reported excellent procedural safety and good outcomes in patients considered inoperable by conventional surgical treatment [17,18]. In a recent study, TTVR through the EVOQUE system demonstrated high rates of procedural success, symptomatic improvement, and survival, and low rates of complications at the two-year follow-up [19]. Although it is still reserved for compassionate use only, and the treatment protocols and the durability of the transcatheter prosthesis are not yet defined, this technique seems promising for those patients where TEER is not feasible [20].

The central focus of our case revolves around confirming the practicality, safety, and effectiveness of implanting a fully percutaneous transcatheter transfemoral tricuspid valve such as the EVOQUE valve system. Additionally, we presented favorable outcomes and a return to an improved functional status during the 18-month follow-up period. In this era of rapid development in transcatheter valve heart disease management, TTVR is expanding the therapeutic options of clinicians, especially in patients who were judged at prohibitive surgical risk.

## Conclusions

The EVOQUE system for TTVR demonstrates excellent mid-term results concerning safety, survival, and improvement in the quality of life for patients categorized as having a prohibitive surgical risk or for those not suitable for TEER. A multidisciplinary team supported by multimodality imaging plays a pivotal role in determining the appropriate therapeutic approach. However, additional research is necessary to refine the best practices for optimal patient management.
